# Radical Cure of Popliteal Aneurism

**Published:** 1883-02

**Authors:** S. Pixley


Radical Cure of Popliteal Aneurism.—Arthur Seeley, twenty-four years of age, single, on the 1st of January, 1882, came to me for medical advice. My diagnosis was Popliteal Aneurism. The following facts were elicited: some eight years ago he had a pair of shears penetrate the popliteal space of his left leg, which confined him to bed for two weeks, soon after which he recovered the use of his leg. Some three years previous to the first of February, 1882, he complained of stiffness of the knee, and a small tumor in the popliteal space. Upon examination, I advised him to consult the professors of the Cleveland City Hospital for treatment.

He entered the hospital on the 21st of February, 1882, and his treatment while there was as follows: First by flexion of the leg upon the thigh, bound by bandages, his heels touched his nates, in which position he was kept for three weeks, his suffering being allayed by morphine, administered hypodermically. There was no improvement of the aneurism. The bandages were removed on the 14th of March, and an attempt was made to straighten the leg, which stood at nearly right angles with the femur.

The leg was raised and placed in an extension sling. An India-rubber bandage was then applied from the calf of the leg to the lower portion of the middle third of the femur, three days, when the swelling and pain of the foot required the removal of the bandage and the re-application of it from the toes to the middle third of the femur.

This treatment was continued until the 4th of April, when he was discharged from the hospital without relief.

The patient returned to this place to have the artery ligated.

On the 11th of April I procured the assistance of Doctors Humphry, Cole and Humphry, to assist me in an attempt to make a radical cure by digital pressure·

At nine of same day we began digital pressure, and continued same for twelve hours, then left patient to rest for thirty-six hours. Again resumed pressure for six hours, at which time pulsation entirely ceased through the aneurism. We still continued pressure for three hours, when we considered the cure effected, which has proved itself permanent.

On July 1st, the tumor was almost obliterated, and the patient resumed his usual work, and has continued well up to December 8, 1882.—*S. Pixley, M.D., in the Medical and Surgical Reporter.*

